# Which is better for adolescents’ health: “regular exercise” or “weekend warriors”? A cross-sectional study based on a sample from Tibet

**DOI:** 10.3389/fpubh.2026.1857690

**Published:** 2026-07-06

**Authors:** Meng Yuan, Feng Peng, Bowen Song, Chenyu Liu, Xiaotong Zhang, Chen Zhang, Yi Sun

**Affiliations:** 1 College of Physical Education, Ludong University, Yantai, Shandong, China; 2 Research Center for Adolescent Exercise Promotion and Health, Ludong University, Yantai, Shandong, China

**Keywords:** children and adolescents, physical activity patterns, physical health, Tibetan, weekend warriors

## Abstract

**Objective:**

This study aimed to analyze the distribution of one-week physical activity patterns among adolescents in Tibet and to investigate the associations of “weekend warrior” and regular activity patterns with physical health.

**Methods:**

This cross-sectional study was conducted from June to December 2020 and included 1,275 Tibetan and Han students aged 12–18 years from two middle schools in Lhasa. After quality control, 1,070 participants met the inclusion criteria and were included in the final analysis. Physical health was assessed using a composite score derived from nine indicators covering body composition, muscular strength, cardiorespiratory endurance, agility and coordination, speed, and flexibility. Among participants who met the recommended weekly MVPA volume, regular and weekend warrior patterns were distinguished according to whether ≥60 min/day of MVPA was accumulated on relatively more or fewer valid wear days. Chi-square tests were used to compare ethnic differences in sex, school stage, and activity patterns. Independent-samples t tests were used to compare socioeconomic status, whereas Mann–Whitney U tests were used to compare sleep quality and dietary habit scores. Binary logistic regression was used to examine the associations between insufficient, regular, and weekend warrior patterns and physical health.

**Results:**

Compared with the insufficient activity pattern, both the regular activity pattern (OR = 2.193, 95% CI: 1.324–3.633, *p* = 0.002) and the weekend warrior pattern (OR = 3.278, 95% CI: 1.386–7.749, *p* = 0.007) were associated with better physical health. No significant difference was observed between the regular and weekend warrior patterns in their associations with physical health (*p* = 0.417). Among adolescents with an insufficient activity pattern, the likelihood of achieving a physical health score of ≥80 increased gradually with increasing activity intensity, as indicated by the proportion of vigorous physical activity (VPA) and metabolic equivalent (MET) values (*p_trend_* < 0.001 and *p_trend_* = 0.006, respectively).

**Conclusion:**

Both regular and weekend warrior patterns were associated with better physical health among adolescents in Tibet. No significant difference was observed between the two patterns, but this finding should be confirmed in future studies with larger weekend warrior samples. Among adolescents with an insufficient activity pattern, activity intensity was also associated with physical health; however, this finding should be interpreted cautiously given the cross-sectional design.

## 1 Introduction

During adolescence, a critical period of growth and development, active participation in physical activity (PA) not only contributes to bone development (1), muscle strength, and cardiorespiratory function, but also helps relieve academic stress and improve emotional well-being. Regular physical activity has been associated with lower risks of cardiovascular disease (2), type 2 diabetes (3), hypertension (4), cancer (5), obesity (6), and depression (7). The World Health Organization recommends that children and adolescents engage in at least 60 min of moderate-to-vigorous physical activity (MVPA) every day throughout the week (8). However, evidence shows that approximately 80% of school-going adolescents worldwide still do not meet the recommended minimum level of physical activity (9).

Under the influence of fast-paced lifestyles and multiple task demands, maintaining regular physical activity remains a challenge for many people. Therefore, some individuals tend to concentrate most of their physical activity on weekends, a pattern commonly referred to as “weekend warriors” (10), that is, completing at least 150 min of MVPA on 1–2 days within a week. Min et al. (11) found that a weekend warrior MVPA pattern within 1–2 days was associated with a lower risk of brain disease. White et al. (12) found in children and adolescents that, when the total amount of MVPA met the recommended level, there was no significant difference in cardiovascular health benefits between regular physical activity and weekend warrior pattern. From the perspective of physical activity composition, the health effects of physical activity do not depend solely on frequency and duration; exercise intensity is also an important indicator influencing health outcomes. Previous studies have shown (13) that vigorous-intensity exercise has greater advantages than moderate-intensity exercise in improving certain health indicators. For example, a lower body fat percentage has been found to be closely associated with vigorous physical activity, whereas no significant association has been observed with moderate-intensity physical activity (14). In addition, Ross et al. (15) further found that, under the same total exercise volume, increasing exercise intensity was associated with further improvements in cardiorespiratory fitness. However, existing studies on the relationships of different one-week activity patterns and exercise intensity with physical health among adolescents remain limited. Tibetan adolescents may represent a distinct population for examining these associations. Tibet is located on the Qinghai–Tibet Plateau and is characterized by a high-altitude environment and unique geographic and sociocultural contexts, which may influence adolescents’ physical activity and physical fitness. Previous studies have reported that children and adolescents living in Tibet at altitudes above 3,500 m exhibit different patterns of moderate-to-vigorous physical activity across weekdays and weekends (16), and their cardiorespiratory fitness levels differ from those reported in peers from plain regions and international reference populations (17). Therefore, evidence from adult populations or adolescents living in low-altitude regions may not be directly generalizable to adolescents living in Tibet.

At present, factors such as heavy academic burdens and extracurricular tutoring (18) may restrict regular participation in physical activity among children and adolescents, making flexible activity arrangements increasingly relevant. However, it remains unclear whether achieving the recommended weekly MVPA volume through a regular pattern or a more concentrated weekend warrior pattern is similarly associated with physical health among adolescents in Tibet. It is also unclear whether activity intensity is associated with physical health among adolescents who do not meet the recommended weekly MVPA volume. Therefore, this study aimed to analyze the distribution of one-week physical activity patterns among adolescents in Tibet and to examine the associations of regular and weekend warrior patterns with physical health. We hypothesized that both regular and weekend warrior patterns would be associated with better physical health compared with an insufficient activity pattern, that no significant difference would be observed between the two sufficient-activity patterns, and that activity intensity would be positively associated with physical health among adolescents with insufficient weekly activity volume.

## 2 Methods

### 2.1 Participants

This cross-sectional study was conducted in Lhasa from June to December 2020. Two middle schools (Lhasa No. 8 Middle School and Lhasa Middle School) were selected as study sites. Tibetan and Han students aged 12–18 years were recruited at the class level using cluster sampling across grades. To account for the imbalance in the number of Tibetan and Han students, Tibetan and Han classes were sampled separately within each grade, with the target sampling ratio set at approximately 3:1. The Tibetan students were long-term plateau residents, and the Han students had lived at high altitude for at least 2 years before participation. The inclusion criteria were as follows: (1) current enrollment in junior or senior high school; (2) age 12–18 years; (3) no physical disabilities or pre-existing health conditions that could affect physical activity participation or physical fitness testing; and (4) voluntary participation with parental or guardian consent. After enrollment, participants were provided with accelerometers and questionnaires to assess daily physical activity (PA), socioeconomic status (SES), dietary habits, and sleep quality, while anthropometric data were collected simultaneously. A total of 1,275 students were initially recruited. After excluding 19 participants with invalid accelerometer data and 186 with incomplete questionnaire data, 1,070 participants were included in the final analysis. The study protocol was approved by the Human Research Ethics Committee of East China Normal University (Approval No. HR 0077-2020).

### 2.2 Physical activity

Physical activity was assessed using the ActiGraph GT3X+ accelerometer. Accelerometer monitoring was conducted mainly from September to November 2020 during the regular school semester. Participants were instructed to wear the device on the right hip for 7 consecutive days, including 5 weekdays and 2 weekend days, and to remove it only during water-based activities such as swimming, bathing, or showering. Before distribution, all devices were initialized using the same settings and checked for battery status, clock time, and recording function. Standardized instructions were provided, and teachers and research staff reminded participants to wear the device during the monitoring period.

Data were collected using a 1-s epoch and summarized as counts per minute for subsequent processing. Non-wear time was identified as at least 60 consecutive minutes of zero counts (19). Following Marques et al. (20), a valid day was defined as at least 600 min of accelerometer wear time, and participants with at least 4 valid days, including 3 weekdays and 1 weekend day, were included in the final analysis. Accelerometer files with device malfunction, abnormal timestamps, or insufficient valid wear days were considered invalid and excluded from analysis. Physical activity intensity was classified using the cut-points proposed by Evenson et al. (21): sedentary behavior (SB; 0–100 counts/min), light-intensity physical activity (LPA; 101–2,295 counts/min), moderate-intensity physical activity (MPA; 2,296–4,011 counts/min), and vigorous-intensity physical activity (VPA; ≥4,012 counts/min). METs were estimated using the equation proposed by Freedson et al. (22): METs = 2.757 + (0.0015 × counts/min) − (0.08957 × age) − (0.000038 × counts/min × age).

### 2.3 Physical health score

In this study, physical health was evaluated using nine indicators covering six domains: body composition, muscular strength, cardiorespiratory endurance, agility and coordination, speed, and flexibility. Different weights were assigned to each indicator. Specifically, BMI and waist circumference were used to assess body composition, with weighting coefficients of 14 and 6%, respectively. Grip strength, 30-s sit-ups, and standing long jump were used to assess muscular strength, with weighting coefficients of 5, 9, and 10%, respectively. Cardiorespiratory endurance was assessed using the 20-m shuttle run test, with a weighting coefficient of 28%. The 20-s side-step test was used to assess agility and coordination, with a weighting coefficient of 8%. Speed was assessed using the 50-m dash, with a weighting coefficient of 8%, and flexibility was assessed using the sit-and-reach test, with a weighting coefficient of 12%. Each indicator was scored on a 100-point scale, and the total physical health score was calculated as the weighted sum of all indicators according to established Chinese children and adolescents’ physical health evaluation standards (23). The weighting coefficients reflect the relative contribution of different physical fitness domains to the overall physical health evaluation. Previous research has shown that this evaluation standard has good scientific validity and feasibility for assessing the physical health status of children and adolescents in China (23). Total scores were classified as excellent (≥90), good (80.0–89.9), passing (60.0–79.9), or failing (<60.0). For the primary logistic regression analyses, participants were divided into high and low physical health groups using a cut-off score of 80, corresponding to the threshold for the “good” level in the established evaluation standard. This classification was consistent with the primary aim of examining whether different physical activity patterns were associated with the likelihood of achieving a favorable physical health level.

### 2.4 Delineation of activity patterns

Activity patterns were classified in two steps. First, participants were classified according to whether they met the recommended weekly volume of moderate-to-vigorous physical activity (MVPA). Based on the World Health Organization recommendation that children and adolescents should accumulate an average of at least 60 min/day of MVPA, a threshold of 420 min/week was used to define sufficient weekly MVPA. Second, among participants who met this weekly MVPA threshold, activity patterns were further classified according to how MVPA was distributed across valid accelerometer wear days. This classification was adapted from the weekly fractionalization framework proposed by Janssen et al. (24), which distinguishes physically active children and youth according to whether the recommended daily MVPA target is achieved on fewer or more days of the week. Therefore, in the present study, regular and weekend warrior patterns differed in the distribution of MVPA across valid wear days, rather than in total weekly MVPA volume.

For participants with fewer than 7 valid accelerometer wear days, weekly MVPA was estimated by multiplying the average daily MVPA across valid wear days by 7. Participants with estimated weekly MVPA <420 min were classified as having an insufficient activity pattern. Participants with estimated weekly MVPA ≥420 min were considered to have met the recommended weekly MVPA volume and were further classified as having either a regular activity pattern or a weekend warrior pattern.

A regular activity pattern was defined as meeting the weekly MVPA threshold and accumulating ≥60 min/day of MVPA on more than the median number of possible valid days within each wear-day category. This indicates that MVPA was distributed across relatively more valid wear days. A weekend warrior pattern was defined as meeting the same weekly MVPA threshold but accumulating ≥60 min/day of MVPA on no more than the median number of possible valid days within each wear-day category. This indicates that MVPA was concentrated on relatively fewer valid wear days.

The median valid-day threshold was used because participants had 4–7 valid accelerometer wear days. Applying a fixed 5-day criterion to all participants would have disadvantaged those with fewer valid wear days, especially participants with only 4 valid wear days. Therefore, the median valid-day threshold was used to adapt the 7-day classification framework to participants with incomplete but valid accelerometer data, while reducing potential misclassification caused by differences in valid wear days (24).

In this study, the term “weekend warrior pattern” was used to describe a concentrated weekly MVPA distribution pattern, rather than physical activity accumulated in continuous bouts or necessarily performed only on weekend days. The specific classification criteria are shown in Table 1.

**Table 1:** Classification criteria for physical activity patterns according to valid accelerometer wear days.

Valid days	Insufficient activity pattern	Regular activity pattern	Weekend warrior pattern
4	Estimated weekly MVPA <420 min	Estimated weekly MVPA ≥420 min and ≥60 min/day MVPA on 3–4 valid days	Estimated weekly MVPA ≥420 min and ≥60 min/day MVPA on 1–2 valid days
5	Estimated weekly MVPA <420 min	Estimated weekly MVPA ≥420 min and ≥60 min/day MVPA on 4–5 valid days	Estimated weekly MVPA ≥420 min and ≥60 min/day MVPA on 1–3 valid days
6	Estimated weekly MVPA <420 min	Estimated weekly MVPA ≥420 min and ≥60 min/day MVPA on 4–6 valid days	Estimated weekly MVPA ≥420 min and ≥60 min/day MVPA on 1–3 valid days
7	Estimated weekly MVPA <420 min	Estimated weekly MVPA ≥420 min and ≥60 min/day MVPA on 5–7 valid days	Estimated weekly MVPA ≥420 min and ≥60 min/day MVPA on 1–4 valid days

Participants were first classified according to whether they met the recommended weekly MVPA volume of 420 min/week. Among those meeting this threshold, regular and weekend warrior patterns were distinguished by the number of valid wear days on which participants accumulated ≥60 min/day of MVPA. For each valid-wear-day category, the median number of possible valid days was used as the cut-off to distinguish activity distributed across more valid days from activity concentrated on fewer valid days.

### 2.5 Socioeconomic status (SES)

Socioeconomic status (SES) was assessed using parental questionnaires covering three dimensions: education, occupation, and income. Parental education was quantified by years of schooling, parental occupation was coded according to the International Standard Classification of Occupations (ISCO), and monthly household income was categorized into four levels with corresponding scores: ≤2,000 CNY (2 points), 2,001–5,000 CNY (5 points), 5,001–8,000 CNY (8 points), and >8,000 CNY (10 points). After data processing and missing-value treatment, all SES-related variables were standardized as z-scores, and principal component analysis (PCA) was performed to derive a composite SES score (25).

### 2.6 Dietary habits

Dietary habits were assessed using a questionnaire that collected information on the number of days per week participants ate breakfast, the number of days per week they consumed at least one egg, the number of days per week they drank at least one cup of milk, yogurt, or soymilk, and the usual daily frequency of sugar-sweetened beverage consumption over the past month. The dietary habits score was calculated using the same procedure as that for SES. Specifically, breakfast consumption, egg consumption, and milk/yogurt/soymilk intake were categorized and scored as ≤2 times/week = 1 point, 3–4 times/week = 2 points, and 5–7 times/week = 3 points. Sugar-sweetened beverage consumption was categorized and scored as 1 time/day or none = 3 points, 2–4 times/day = 2 points, and ≥5 times/day = 1 point. After variable screening or transformation and missing-value processing, all variables were standardized and entered into a principal component analysis to derive the dietary habits score.

### 2.7 Sleep quality

Sleep quality was assessed using the Pittsburgh Sleep Quality Index (PSQI) (26). The PSQI consists of 19 items and can be divided into seven components: subjective sleep quality, sleep latency, sleep duration, sleep efficiency, sleep disturbances, use of hypnotic medication, and daytime dysfunction. Each component is scored on a scale of 0–3, and the sum of the component scores constitutes the total PSQI score, with higher scores indicating poorer sleep quality. PSQI scores were categorized as 0–5, very good; 6–10, okay; 11–15, fair; and 16–21, very poor.

### 2.8 Statistical analysis

Categorical variables were presented as frequencies and percentages, and continuous variables were presented as mean ± standard deviation or median (interquartile range), as appropriate. The chi-square test was used to compare ethnic differences in sex, school stage, and activity patterns. Independent-samples t tests were used to compare ethnic differences in socioeconomic status, whereas Mann–Whitney *U* tests were used to compare sleep quality scores and dietary habits scores because these variables were not normally distributed. For the regression analyses, physical health was dichotomized using a cut-off score of 80. Binary logistic regression was performed to examine the associations between insufficient, regular, and weekend warrior patterns and physical health. Model 1 was unadjusted, whereas Model 2 was adjusted for sex, age, socioeconomic status, dietary habits, and sleep quality. Odds ratios (ORs) and 95% confidence intervals (CIs) were reported, and a two-sided *p* value <0.05 was considered statistically significant. Given the cross-sectional design of this study, all regression results were interpreted as associations rather than evidence of causal relationships. For trend analyses, quartile categories of MVPA duration, VPA proportion, and MET values were coded as ordinal variables from 1 to 4 and entered into the logistic regression models as continuous variables. Model performance was evaluated using the omnibus test of model coefficients and the Hosmer–Lemeshow goodness-of-fit test. Multicollinearity among covariates was assessed using variance inflation factors (VIFs). For subgroup models with sparse data or unstable estimates, non-estimable *ORs* were not reported.

## 3 Results

Table 2 presents participant characteristics and the distribution of activity patterns. Compared with Tibetan adolescents, Han Chinese adolescents had significantly higher SES and sleep quality scores but lower dietary habits scores (all *p* < 0.001). They also showed a higher proportion of the insufficient activity pattern (90.2% vs. 83.5%) but lower proportions of the regular (7.0% vs. 11.8%) and weekend warrior (2.8% vs. 4.7%) activity patterns, with a statistically significant difference (*p* = 0.006).

**Table 2:** Participant characteristics and distribution of activity patterns.

	Han Chinese		Tibetan		*χ*^2^/T/Z	*p*
	Number	Percentage	Number	Percentage		
Gender					1.363	0.243
Male	226	48.1%	267	44.5%		
Female	244	51.9%	333	55.5%		
School stage					7.734	0.005
Junior high school	156	33.2%	249	41.5%		
High school	314	66.8%	351	58.5%		
Socioeconomic status	1.42 ± 2.41		−1.48 ± 4.04		14.456	<0.001
Sleep quality score	4.00 (2.00, 6.00)		3.00 (2.00, 5.00)		−3.793	<0.001
Dietary habits score	−0.51(−0.82, 0.43)		0.43(−0.51, 1.38)		−7.903	<0.001
Activity pattern					10.137	0.006
Insufficient activity pattern	424	90.2%	501	83.5%		
Regular activity pattern	33	7.0%	71	11.8%		
Weekend warrior pattern	13	2.8%	28	4.7%		

Table 3 shows the associations between activity patterns and physical health among adolescents in Tibet. Using the insufficient activity pattern as the reference, adolescents with regular and weekend warrior patterns were more likely to achieve a physical health score of 80 or above (regular: OR = 2.19, 95% CI: 1.32–3.63, *p* = 0.002; weekend warrior: OR = 3.28, 95% CI: 1.39–7.75, *p* = 0.007). When the weekend warrior pattern was used as the reference, the regular activity pattern was not significantly associated with physical health (OR = 0.67, 95% CI: 0.25–1.77, *p* = 0.417).

**Table 3:** Relationship between activity patterns and physical health among adolescents in Tibet.

Activity pattern	Model 1		Model 2	
	OR (95% CI)	*p*-value	OR (95% CI)	*p*-value
Using the “insufficient” activity pattern as the reference				
“Insufficient”	Ref	–	Ref	–
“Regular”	1.90 (1.26, 2.86)	0.002	2.19 (1.32, 3.63)	0.002
“Weekend warrior”	3.14 (1.62, 6.07)	0.001	3.28 (1.39, 7.75)	0.007
Using the “weekend warrior” activity pattern as the reference				
“Weekend warrior”	Ref	–	Ref	–
“Insufficient”	0.32 (0.17, 0.62)	0.001	0.31(0.13, 0.72)	0.007
“Regular”	0.61 (0.29, 1.28)	0.190	0.67(0.25, 1.77)	0.417

Table 4 shows the associations between MVPA duration and physical health across different activity patterns. In the insufficient activity pattern group, higher MVPA duration was associated with a greater likelihood of achieving a physical health score of 80 or above (*p_trend_* = 0.001). In contrast, no significant association was observed between MVPA duration and physical health in either the regular (*p_trend_* = 0.567) or weekend warrior (*p_trend_* = 0.559) activity pattern groups.

**Table 4:** Relationship between MVPA and physical health in different activity patterns.

Activity pattern	*n* (%)	Model 1		Model 2	
		OR (95% CI)	*p*-value	OR (95% CI)	*p*-value
“Insufficient” activity pattern					
Q1 (≤36.99 min/day)	231 (24.97%)	Ref	–	Ref	–
Q2 (36.99–46.17 min/day)	232 (25.08%)	2.67 (1.76, 4.03)	<0.001	1.85 (1.15, 2.96)	0.011
Q3 (46.17–53.90 min/day)	231 (24.97%)	2.69 (1.78, 4.06)	<0.001	1.94 (1.21, 3.12)	0.006
Q4 (≥53.90 min/day)	231 (24.97%)	4.02 (2.66, 6.06)	<0.001	2.29 (1.42, 3.67)	<0.001
*p_trend_*			<0.001		0.001
“Regular” activity pattern					
Q1 (≤72.70 min/day)	26 (25.00%)	Ref	–	Ref	–
Q2 (72.70–78.73 min/day)	26 (25.00%)	2.18 (0.72, 6.61)	0.168	1.48 (0.32, 6.93)	0.618
Q3 (78.73–87.13 min/day)	26 (25.00%)	1.86 (0.62, 5.59)	0.269	1.02 (0.26, 4.00)	0.980
Q4 (≥87.13 min/day)	26 (25.00%)	1.59 (0.53, 4.76)	0.406	1.72 (0.33, 8.98)	0.518
*p_trend_*			0.484		0.567
“Weekend warrior” activity pattern					
Q1 (≤61.75 min/day)	10 (24.39%)	Ref	–	Ref	–
Q2 (61.75–64.08 min/day)	10 (24.39%)	1.00 (0.17, 5.99)	1.000	–	–
Q3 (64.08–70.91 min/day)	11 (26.83%)	1.78 (0.28, 11.12)	0.538	–	–
Q4 (≥70.91 min/day)	10 (24.39%)	1.56 (0.24, 9.91)	0.640	–	–
*p_trend_*			0.524		0.559

Within each activity pattern group, MVPA duration was categorized into quartiles (Q1–Q4). The dependent variable was achieving a physical health score of ≥80 points. ORs and 95% CIs were estimated using logistic regression models. *p_trend_* was calculated by entering the quartile variable as an ordinal continuous variable in the logistic regression model. “–” indicates that the OR was not estimated because of sparse data or unstable model estimates.

Table 5 shows the associations between VPA proportion and physical health across different activity patterns. In the insufficient activity pattern group, a higher VPA proportion was associated with a greater likelihood of achieving a physical health score of 80 or above (*p_trend_* < 0.001). In contrast, no significant association was observed between VPA proportion and physical health in either the regular (*p_trend_* = 0.828) or weekend warrior (*p_trend_* = 0.198) activity pattern groups.

**Table 5:** Associations between VPA proportion and physical health across different activity patterns.

Activity pattern	*n* (%)	Model 1		Model 2	
		OR (95% CI)	*p*-value	OR (95% CI)	*p*-value
“Insufficient” activity pattern					
Q1 (≤29.92%)	231 (24.97%)	Ref	–	Ref	–
Q2 (29.92–35.86%)	232 (25.08%)	1.14 (0.77, 1.67)	0.515	1.68 (1.08, 2.60)	0.021
Q3 (35.85–41.90%)	231 (24.97%)	1.17 (0.79, 1.71)	0.434	2.00 (1.26, 3.18)	0.003
Q4 (≥41.90%)	231 (24.97%)	1.82 (1.25, 2.66)	0.002	2.42 (1.56, 3.77)	<0.001
*p_trend_*			0.002		<0.001
“Regular” activity pattern					
Q1 (≤39.66%)	26 (25.00%)	Ref	–	Ref	–
Q2 (39.66–45.83%)	26 (25.00%)	0.86 (0.29, 2.55)	0.781	1.46 (0.33, 6.43)	0.614
Q3 (45.83–51.25%)	26 (25.00%)	1.37 (0.46, 4.14)	0.575	0.89 (0.20, 4.01)	0.876
Q4 (≥51.25%)	26 (25.00%)	0.86 (0.29, 2.55)	0.781	0.63 (0.13, 3.11)	0.568
*p_trend_*			1.000		0.828
“Weekend warrior” activity pattern					
Q1 (≤39.29%)	10 (24.39%)	Ref	–	Ref	–
Q2 (39.29–42.26%)	10 (24.39%)	1.71 (0.22, 13.41)	0.608	–	–
Q3 (42.26–49.24%)	11 (26.83%)	0.51 (0.09, 3.11)	0.469	–	–
Q4 (≥49.24%)	10 (24.39%)	0.64 (0.10, 4.10)	0.640	–	–
*p_trend_*			0.406		0.198

Within each activity pattern group, VPA proportion was categorized into quartiles (Q1–Q4). The dependent variable was achieving a physical health score of ≥80 points. ORs and 95% CIs were estimated using logistic regression models. *p_trend_* was calculated by entering the quartile variable as an ordinal continuous variable in the logistic regression model. “–” indicates that the OR was not estimated because of sparse data or unstable model estimates.

Table 6 shows the associations between MET values and physical health across different activity patterns. In the insufficient activity pattern group, higher MET values were associated with a greater likelihood of achieving a physical health score of 80 or above (*p_trend_* = 0.006). In contrast, no significant association was observed between MET values and physical health in either the regular (*p_trend_* = 0.205) or weekend warrior (*p_trend_* = 0.531) activity pattern groups.

**Table 6:** Associations between MET values and physical health across different activity patterns.

Activity pattern	*n* (%)	Model 1		Model 2	
		OR (95% CI)	*p*-value	OR (95% CI)	*p*-value
“Insufficient” activity pattern					
Q1 (≤1.17)	222 (24.00%)	Ref	–	Ref	–
Q2 (1.17–1.21)	266 (28.76%)	0.93 (0.64, 1.34)	0.679	0.98 (0.64, 1.50)	0.920
Q3 (1.21–1.24)	193 (20.86%)	1.30 (0.88, 1.92)	0.196	1.61 (1.00, 2.58)	0.050
Q4 (≥1.24)	244 (26.38%)	0.85 (0.58, 1.24)	0.387	1.56 (0.97, 2.49)	0.065
*p_trend_*			0.732		0.006
“Regular” activity pattern					
Q1 (≤1.31)	24 (23.08%)	Ref	–	Ref	–
Q2 (1.31–1.34)	27 (25.96%)	1.23 (0.41, 3.74)	0.714	0.92 (0.23, 3.77)	0.911
Q3 (1.34–1.41)	28 (26.92%)	0.64 (0.21, 1.90)	0.417	1.86 (0.26, 13.52)	0.541
Q4 (≥1.41)	25 (24.04%)	1.27 (0.41, 3.94)	0.680	2.86 (0.41, 19.76)	0.288
*p_trend_*			0.989		0.205
“Weekend warrior” activity pattern					
Q1 (≤1.26)	10 (24.39%)	Ref	–	Ref	–
Q2 (1.26–1.31)	14 (34.15%)	1.67 (0.30, 9.27)	0.560	–	–
Q3 (1.31–1.32)	6 (14.63%)	0.67 (0.09, 5.13)	0.697	–	–
Q4 (≥1.32)	11 (26.83%)	1.78 (0.28, 11.12)	0.538	–	–
*p_trend_*			0.741		0.531

Within each activity pattern group, MET values were categorized into quartiles (Q1–Q4). The dependent variable was achieving a physical health score of ≥80 points. ORs and 95% CIs were estimated using logistic regression models. *p_trend_* was calculated by entering the quartile variable as an ordinal continuous variable in the logistic regression model. “–” indicates that the OR was not estimated because of sparse data or unstable model estimates.

It should be noted that the weekend warrior subgroup was relatively small (*n* = 41), particularly after further stratification into quartiles. Therefore, the subgroup-specific estimates for the weekend warrior pattern in Tables 4–6 had limited statistical precision and should be interpreted cautiously.

## 4 Discussion

This study found that, compared with the insufficient activity pattern, both regular and weekend warrior patterns were associated with better physical health among adolescents, with no significant difference observed between the two patterns. Among adolescents with an insufficient activity pattern, activity intensity was also associated with physical health; however, this finding should be interpreted cautiously given the cross-sectional design.

This study showed that, in their associations with physical health, no significant difference was observed between adopting a regular weekly physical activity pattern and adopting a weekend warrior pattern. This finding suggests that, compared with activity frequency, the total volume of physical activity may be more closely associated with physical health among adolescents. A prospective cohort study conducted by researchers from the Massachusetts Institute of Technology and Harvard University showed that, as long as the total amount of physical activity recommended by health guidelines was achieved, both regular physical activity and a weekend warrior pattern were associated with reduced risks of 264 diseases, with the strongest association observed for cardiometabolic diseases (27)^.^ This result is consistent with the findings of White et al. (12) in children and adolescents, and similar conclusions have also been reported in adult studies. Lee et al. (10) found that, regardless of the frequency of physical activity, as long as energy expenditure through physical activity reached 1,000 kcal per week, mortality risk could be significantly reduced among individuals who were otherwise at low risk. Shiroma et al. (28) further reported that even “weekend warriors” who were active only 1–2 days per week had a significantly reduced risk of mortality, with reductions comparable to those observed in consistently active individuals. O’Donovan et al. (29), based on survey data from more than 63,000 adults in England and Scotland, also showed that, compared with insufficiently active individuals, “weekend warriors” had lower risks of all-cause, cardiovascular, and cancer mortality, and that the magnitude of these benefits was similar to that observed in frequently active individuals. Taken together, these studies suggest that, when a sufficient total volume of physical activity is achieved, different distributions of activity may show similar associations with health outcomes. In the present study, the absence of a significant difference between regular and weekend warrior patterns may therefore be related to the fact that both groups achieved the recommended weekly MVPA volume. However, this finding should not be interpreted as evidence that concentrating activity on fewer days has the same causal effect as regular activity. Adolescents who were able to achieve sufficient weekly MVPA, whether regularly or in a more concentrated pattern, may already have had better physical fitness, stronger exercise habits, or more opportunities to participate in physical activity. Therefore, for children and adolescents, the present findings suggest that weekly MVPA volume is an important dimension to consider, but the temporal direction of this association requires confirmation in longitudinal studies.

This study further found that, for adolescents who have difficulty achieving the recommended total volume of physical activity, activity intensity may be another dimension associated with physical health among adolescents who do not meet the recommended total volume of physical activity. Specifically, in the insufficient activity pattern group, the likelihood of achieving a physical health score of 80 or above increased with higher levels of VPA and MET. This suggests that, under real-life constraints on available activity time, exercise intensity may represent an important dimension associated with physical health, and also indicates the need to encourage adolescents to participate actively in physical activity and gradually achieve the recommended activity level (30). This finding is also consistent with previous studies. Ross et al. (15) found that, under conditions of equal total exercise volume, increasing exercise intensity was associated with further improvements in cardiorespiratory fitness and reductions in abdominal obesity and cardiometabolic risk. Hansen et al. (31) also reported that sustained moderate-to-vigorous physical activity was related to improved glucose metabolism and skeletal muscle oxidative capacity. Taken together, these studies are consistent with the possibility that activity intensity may be another dimension associated with physical health among adolescents who do not meet the recommended weekly MVPA volume. Nevertheless, this association should be interpreted cautiously. Adolescents with better physical fitness may be more capable of engaging in vigorous or higher-intensity activity, and participation in organized sports, structured training, or school-based physical education may be related to both higher activity intensity and better physical health performance. Therefore, the present findings do not indicate that exercise intensity can replace sufficient activity time or causally offset insufficient activity duration. From a practical perspective, schools may consider both activity time and effective exercise load when promoting physical activity among children and adolescents (32). In recent years, China has also emphasized optimizing school physical education and recess arrangements to ensure daily opportunities for student physical activity and to support physical activity participation and health promotion (33–35). Therefore, physical education classes, after-school activities, and recess periods may provide practical settings for supporting students in accumulating moderate-to-vigorous physical activity more effectively within a limited time.

The strength of this study lies in its relatively comprehensive perspective, as it considered physical activity duration, frequency, and intensity in relation to physical health. This multidimensional design helps to describe the complex relationship between physical activity and physical health. However, several limitations should be noted. First, because of the cross-sectional design, this study cannot determine temporal or causal relationships between activity patterns, activity intensity, and physical health, and reverse causality is possible. Second, although several covariates were adjusted for and some potential sources of bias were reduced through participant screening and study design, residual confounding from unmeasured individual or contextual factors cannot be completely excluded. Age was adjusted for in the regression models and may partly account for developmental differences among adolescents, although residual maturational differences may still remain. Third, the weekend warrior pattern appears to be a relatively uncommon activity pattern among children and adolescents. This may be partly related to the school-week structure of adolescents, as most students attend school on weekdays and may have limited opportunities to accumulate the recommended weekly MVPA volume within only a few days. Consistent with this real-world feature of adolescent activity behavior, the weekend warrior subgroup in the present study was relatively small. As a result, the quartile-based subgroup analyses within this pattern had limited statistical power and produced some unstable estimates. Therefore, these subgroup findings should be interpreted as exploratory and with caution. Fourth, although accelerometers provide objective measurements of physical activity, hip-worn devices may not fully capture some activities, such as swimming, cycling, or upper-body movement. Finally, the study population was limited to adolescents in Tibet, which may restrict the generalizability of the findings. Future studies with larger and more diverse samples and longitudinal or intervention designs are needed to further validate these findings.

## 5 Conclusion

Both regular and weekend warrior patterns were associated with better physical health among adolescents in Tibet. No significant difference was observed between the two patterns, but this finding should be confirmed in future studies with larger weekend warrior samples. Among adolescents with an insufficient activity pattern, activity intensity was also associated with physical health; however, this finding should be interpreted cautiously given the cross-sectional design.

## Data Availability

The raw data supporting the conclusions of this article will be made available by the authors, without undue reservation.

## References

[1] Janz KF Letuchy EM Eichenberger Gilmore JM Burns TL Torner JC Willing MC . Early physical activity provides sustained bone health benefits later in childhood. Med Sci Sports Exerc. (2010) 42:1072–8. doi: 10.1249/mss.0b013e3181c619b2, 19997029 PMC2874089

[2] Wernhart S Dinic M Pressler A Halle M. Prävention kardiovaskulärer Erkrankungen durch sport und körperliche Aktivität: Eine Frage der Intensität? [Prevention of cardiovascular diseases through sport and physical activity: a question of intensity?]. Herz. (2015) 40:361–8. doi: 10.1007/s00059-015-4216-4 25804555

[3] Smith AD Crippa A Woodcock J Brage S. Physical activity and incident type 2 diabetes mellitus: a systematic review and dose-response meta-analysis of prospective cohort studies. Diabetologia. (2016) 59:2527–45. doi: 10.1007/s00125-016-4079-0, 27747395 PMC6207340

[4] Shariful Islam M Fardousi A Sizear MI Rabbani MG Islam R Saif-Ur-Rahman KM. Effect of leisure-time physical activity on blood pressure in people with hypertension: a systematic review and meta-analysis. Sci Rep. (2023) 13:10639. doi: 10.1038/s41598-023-37149-2, 37391436 PMC10313796

[5] McTiernan A Friedenreich CM Katzmarzyk PT Powell KE Macko R Buchner D . Physical activity in cancer prevention and survival: a systematic review. Med Sci Sports Exerc. (2019) 51:1252–61. doi: 10.1249/MSS.0000000000001937, 31095082 PMC6527123

[6] Calabrese FM Disciglio V Franco I Sorino P Bonfiglio C Calabrese F . A low glycemic index Mediterranean diet combined with aerobic physical activity rearranges the gut microbiota signature in NAFLD patients. Nutrients. (2022) 14:1773. doi: 10.3390/nu14091773, 35565740 PMC9101735

[7] Warburton DE Nicol CW Bredin SS. Health benefits of physical activity: the evidence. CMAJ. (2006) 174:801–9. doi: 10.1503/cmaj.051351 16534088 PMC1402378

[8] World Health Organization. WHO Guidelines on Physical Activity and Sedentary Behaviour. Geneva: World Health Organization; (2020). Available online at: https://www.who.int/publications/i/item/9789240015128 (accessed April 27, 2021)

[9] Guthold R Stevens GA Riley LM Bull FC. Global trends in insufficient physical activity among adolescents: a pooled analysis of 298 population-based surveys with 1.6 million participants. Lancet Child Adolesc Health. (2020) 4:23–35. doi: 10.1016/s2352-4642(19)30323-2, 31761562 PMC6919336

[10] Lee IM Sesso HD Oguma Y Paffenbarger RS Jr. The "weekend warrior" and risk of mortality. Am J Epidemiol. (2004) 160:636–41. doi: 10.1093/aje/kwh274 15383407

[11] Min J Cao Z Duan T Wang Y Xu C. Accelerometer-derived 'weekend warrior' physical activity pattern and brain health. Nat Aging. (2024) 4:1394–402. doi: 10.1038/s43587-024-00688-y, 39169268

[12] White DA Willis EA Ptomey LT Gorczyca AM Donnelly JE. Weekly frequency of meeting the physical activity guidelines and cardiometabolic health in children and adolescents. Med Sci Sports Exerc. (2022) 54:106–12. doi: 10.1249/MSS.0000000000002767, 34334716 PMC8678143

[13] Zhou X Li J Jiang X. Effects of different types of exercise intensity on improving health-related physical fitness in children and adolescents: a systematic review. Sci Rep. (2024) 14:14301. doi: 10.1038/s41598-024-64830-x, 38906965 PMC11192957

[14] Gutin B Yin Z Humphries MC Barbeau P. Relations of moderate and vigorous physical activity to fitness and fatness in adolescents. Am J Clin Nutr. (2005) 81:746–50. doi: 10.1093/ajcn/81.4.746 15817847

[15] Ross R Hudson R Stotz PJ Lam M. Effects of exercise amount and intensity on abdominal obesity and glucose tolerance in obese adults: a randomized trial. Ann Intern Med. (2015) 162:325–34. doi: 10.7326/M14-1189, 25732273

[16] Nie M Fan C Sun R Wang J Feng Q Zhang Y . Accelerometer-measured physical activity in children and adolescents at altitudes over 3500 meters: a cross-sectional study in Tibet. Int J Environ Res Public Health. (2019) 16:686. doi: 10.3390/ijerph16050686, 30813580 PMC6427613

[17] Fan C Sun R Nie M Wang M Yao Z Feng Q . The cardiorespiratory fitness of children and adolescents in Tibet at altitudes over 3,500 meters. PLoS One. (2021) 16:e0256258. doi: 10.1371/journal.pone.0256258, 34411164 PMC8375997

[18] Brown SL Nobiling BD Teufel J Birch DA. Are kids too busy?: early adolescents' perceptions of discretionary activities, overscheduling, and stress. J Sch Health. (2011) 81:574–80. doi: 10.1111/j.1746-1561.2011.00629.x, 21831071

[19] Chinapaw MJM de Niet M Verloigne M De Bourdeaudhuij I Brug J Altenburg TM. From sedentary time to sedentary patterns: accelerometer data reduction decisions in youth. PLoS One. (2014) 9:e111205. doi: 10.1371/journal.pone.0111205, 25369021 PMC4219709

[20] Marques A Santos R Ekelund U Sardinha LB. Association between physical activity, sedentary time, and healthy fitness in youth. Med Sci Sports Exerc. (2015) 47:575–80. doi: 10.1249/MSS.0000000000000426, 24977696

[21] Evenson KR Catellier DJ Gill K Ondrak KS McMurray RG. Calibration of two objective measures of physical activity for children. J Sports Sci. (2008) 26:1557–65. doi: 10.1080/02640410802334196, 18949660

[22] Freedson P Pober D Janz KF. Calibration of accelerometer output for children. Med Sci Sports Exerc. (2005) 37:S523–30. doi: 10.1249/01.mss.0000185658.28284.ba 16294115

[23] Ji L Yin X Wu H Yang X Liu Y. An exploratory study on the physical health evaluation standards of Chinese children and adolescents under the background of “integration of sports and education”. Sports Sci. (2021) 41:42–54. doi: 10.16469/j.css.202103006

[24] Janssen I Wong SL Colley R Tremblay MS. The fractionalization of physical activity throughout the week is associated with the cardiometabolic health of children and youth. BMC Public Health. (2013) 13:554. doi: 10.1186/1471-2458-13-554, 23742137 PMC3682871

[25] Guo Y Yin X Sun Y Zhang T Li M Zhang F . Research on environmental influencing factors of overweight and obesity in children and adolescents in China. Nutrients. (2021) 14:35. doi: 10.3390/nu14010035 35010910 PMC8746339

[26] Buysse DJ Reynolds CF 3rd Monk TH Berman SR Kupfer DJ. The Pittsburgh Sleep Quality Index: a new instrument for psychiatric practice and research. Psychiatry Res. (1989) 28:193–213. doi: 10.1016/0165-1781(89)90047-4, 2748771

[27] Kany S Al-Alusi MA Rämö JT Pirruccello JP Churchill TW Lubitz SA . Associations of "weekend warrior" physical activity with incident disease and cardiometabolic health. Circulation. (2024) 150:1236–47. doi: 10.1161/circulationaha.124.068669, 39324186 PMC11803568

[28] Shiroma EJ Lee IM Schepps MA Kamada M Harris TB. Physical activity patterns and mortality: the weekend warrior and activity bouts. Med Sci Sports Exerc. (2019) 51:35–40. doi: 10.1249/MSS.0000000000001762, 30138219 PMC6295264

[29] O'Donovan G Lee IM Hamer M Stamatakis E. Association of "weekend warrior" and other leisure time physical activity patterns with risks for all-cause, cardiovascular disease, and cancer mortality. JAMA Intern Med. (2017) 177:335–42. doi: 10.1001/jamainternmed.2016.8014 28097313

[30] Bevington F Piercy KL Olscamp K Hilfiker SW Fisher DG Barnett EY. The move your way campaign: encouraging contemplators and families to meet the recommendations from the physical activity guidelines for Americans. J Phys Act Health. (2020) 17:397–403. doi: 10.1123/jpah.2019-0395 32050160

[31] Hansen D Dendale P Jonkers RA Beelen M Manders RJ Jonkers RAM . Continuous low- to moderate-intensity exercise training is as effective as moderate- to high-intensity exercise training at lowering blood HbA(1c) in obese type 2 diabetes patients. Diabetologia. (2009) 52:1789–97. doi: 10.1007/s00125-009-1354-3, 19370339 PMC2723667

[32] Su J Ji L. An exploration of the concept of exercise density based on the Chinese healthy physical education curriculum model. J Cap Univ Phys Educ Sports. (2019) 31:406–16.

[33] General Office of the State Council Outline of the Planning for Building an Education power (2024–2035) Beijing General Office of the State Council (2025). Available online at: https://www.moe.gov.cn/jyb_xxgk/moe_1777/moe_1778/202501/t20250119_1176193.html (accessed February 27, 2025)

[34] Beijing Municipal Education Commission Notice on Optimizing recess Activities in Compulsory Education Schools Beijing Beijing Municipal Education Commission (2024). Available online at: https://jw.beijing.gov.cn/xxgk/2024zcwj/2024qtwj/202408/t20240829_3784438.html (accessed February 18, 2025)

[35] Ministry of Education. Establishing a long-term Mechanism for regular Supervision and Inspection. Beijing: Ministry of Education (2023).

